# Prenatal Immunity and Influences on Necrotizing Enterocolitis and Associated Neonatal Disorders

**DOI:** 10.3389/fimmu.2021.650709

**Published:** 2021-04-21

**Authors:** Maame Efua S. Sampah, David J. Hackam

**Affiliations:** Division of Pediatric Surgery, Department of Surgery, Johns Hopkins University School of Medicine , Baltimore, MD, United States

**Keywords:** NEC = necrotizing enterocolitis, TL4 – Toll-like receptor 4, microbiota (microorganism), prematurity and low birth weight, pediatric sepsis

## Abstract

Prior to birth, the neonate has limited exposure to pathogens. The transition from the intra-uterine to the postnatal environment initiates a series of complex interactions between the newborn host and a variety of potential pathogens that persist over the first few weeks of life. This transition is particularly complex in the case of the premature and very low birth weight infant, who may be susceptible to many disorders as a result of an immature and underdeveloped immune system. Chief amongst these disorders is necrotizing enterocolitis (NEC), an acute inflammatory disorder that leads to necrosis of the intestine, and which can affect multiple systems and have the potential to result in long term effects if the infant is to survive. Here, we examine what is known about the interplay of the immune system with the maternal uterine environment, microbes, nutritional and other factors in the pathogenesis of neonatal pathologies such as NEC, while also taking into consideration the effects on the long-term health of affected children.

## Introduction

The characteristic underdevelopment of the neonatal immune system predisposes infants to inflammatory disorders, including necrotizing enterocolitis (NEC), an acute inflammatory disease that develops in up to 10% of premature infants (1). NEC is characterized by the sudden development of inflammation and necrosis of the intestine leading to overwhelming sepsis and death in up to half of all patients (2, 3). Unique characteristics of the neonatal immune system that confer a susceptibility to the development of diseases like NEC include the potential exposure of priming antigens within the maternal uterine environment (4), changes in cytokine, growth factor and hormone signaling pathways (5, 6), nutritional effects related to exposure to probiotics and breastmilk (7), as well as patterns of microbial colonization in the gut and other mucosa that are specific to the neonatal period (8). These changes may have long-term effects on the immune system, creating immune activation or tolerance that determine whether pathology will develop (9). Importantly, NEC and other inflammatory neonatal conditions typically also affect the lungs and brain (10–12) resulting in long-term sequelae that may be underappreciated initially. In infants who are fortunate to survive NEC, lifelong problems may develop, including short bowel syndrome (13), nutritional deficiencies impact growth (14), and persistent white matter injury associated with cognitive impairment (10, 15–17) among others.

While many important human clinical studies, as well as preclinical studies in clinically relevant animal models (18), have made important gains in elucidating the complexities of the cellular and biological processes associated with immunological disorders including NEC, many gaps in knowledge in this field remain. We will review available studies that explore the pathogenesis of neonatal gut inflammation and its short- and long-term impacts on various secondary organ systems. We will assess existing research gaps, and the potential future directions for investigations in the field.

## Impaired Immunity in the Neonate

Impairment of many components of the immune system is well documented in neonates and is especially exacerbated in preterm infants (19). This is due to a relative lack of antigenic exposure and enhanced self-modulatory immunosuppressive mechanisms that make way for beneficial microbial colonization and prevent potentially harmful inflammation and autoimmunity (19). As a result, preterm infants are exposed to increased risk of infections from a variety of potential pathogens (20, 21). Known deficiencies include decreased physical integrity of the pulmonary and gastrointestinal epithelial barriers (22, 23), reduced numbers of goblet cells and thinner mucus secretions (24, 25), low numbers of Paneth cells with decreased antimicrobial producing function (26), lower levels of circulating maternal IgG with low opsonic activity (27–29), complement protein deficiency in the setting of reduced activity (30–32), and reduction in numbers and function of neutrophils and monocytes (33–36). As a result of these characteristics, multiple levels of susceptibility to bacterial, viral and fungal infection exist. The implications of these impairments in immunity are reflected in data from clinical studies that report up to five-fold higher risk of sepsis in preterm infants compared with their term counterparts (37, 38). Preterm infants diagnosed with sepsis are, in turn, at significantly higher risk of respiratory and neurological complications such as respiratory distress syndrome, severe intraventricular hemorrhage and periventricular leukomalacia (39). These findings contribute considerably to prolonged neonatal hospital stays (40), increased numbers of rehospitalizations and overall mortality compared with term infants (41, 42).

Deficiencies that allow pathogenic invasion from colonized sites such as the respiratory and gastrointestinal tract in preterm infants are compounded by a tendency toward exaggerated immune activity (43–45). Certain components of the immune system have been noted to be reach a heightened degree of activation, resulting in pathogenic inflammation that has severe ramifications for disease pathogenesis. For instance, Th17 cells are abundant lymphocytes at the intestinal mucosa of the premature newborn (46) and contribute to host defense against extracellular microbial pathogens (47, 48). This subset of CD4+ T cells mainly produce IL-17, which has been shown to induce a pro-inflammatory state, leading to infection-induced immunopathology in many human autoimmune diseases, including multiple sclerosis, rheumatoid arthritis, psoriasis and Lyme arthritis (49–51). In the preterm infant, the relative abundance of IL-17 signaling dampens the effect of counter-inflammatory mechanisms such as Foxp3^+^ Treg cell activity and in fact, downregulates Foxp3^+^ Treg expression (52). The net result of this skew towards a pro-inflammatory lymphocyte state is the finding of impaired enterocyte tight junctions, increased enterocyte apoptosis, and decreased enterocyte proliferation, culminating in global mucosal injury as observed in necrotizing enterocolitis (46).

## Innate Immune Signaling and Downstream Consequences

The exaggerated signaling in response to TLR4 in the premature infant represents an important example of immature immune activation that leads to disease (53). The cell walls of gram-negative bacteria like *Escherichia coli* and *Helicobacter pylori* contain endotoxins which mediate host recognition and inflammatory response to infection. Lipopolysaccharide (LPS), which is the main endotoxin encountered, binds to an intramembrane complex made up of TLR4 and CD14 and initiates the recruitment of myeloid differentiation primary response gene 88 (MyD88), signaling an inflammatory response involving the activation of nuclear factor-κB (54). This response is accompanied by cellular stress responses, leading to the release of pro-inflammatory cytokines including IL-6, IL-1β, TNF-α and other cellular stress markers including nitric oxide (54), as well as the expression of co-stimulatory molecules (55). It should be pointed out that other TLR4 ligands exist, including viral proteins, endogenous proteins such as low-density lipoprotein, beta-defensins, and heat shock proteins (56–59). TLR4 signaling may also occur *via* an alternative adapter protein TRIF (60, 61). In leukocytes, the downstream effects of TLR4 activation are critical for host defense against infection (62, 63) *via* NF-κB activation and downstream cytokine-mediated responses (63). However, in the gut, TLR4 is notably an essential component for normal intestinal development *via* the Notch signaling pathway (64, 65). For this reason, TLR4 is expressed at higher levels in the premature (and thus still developing) gut as compared to the full-term gut, which does not result in an inflammatory response given the bacteria-free environment of the developing fetus. By contrast, when the premature infant is born and exposed to the microbiota of the environment, colonization of the intestine occurs, leading to activation of TLR4 by LPS, whereupon TLR4 switches from a developmental to an inflammatory role, leading to the induction of NEC.

**Figure 1:**
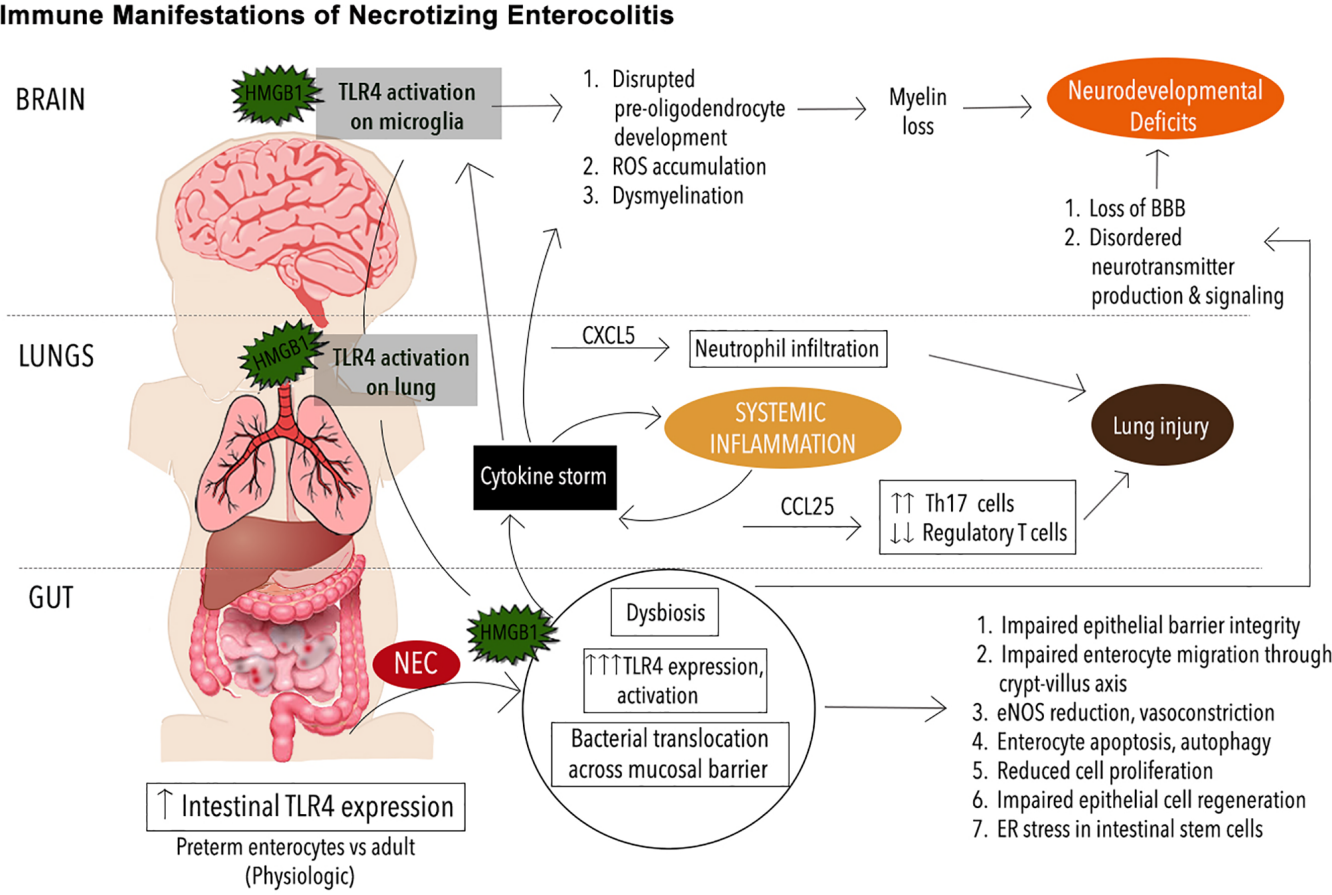
Immune Manifestations in NEC. Schematic illustrating immune signaling involving toll-like receptor 4 (TRL4) in necrotizing enterocolitis (NEC) pathogenesis. The premature immune system leaves the neonate prone to infectious and inflammatory diseases such as NEC. Mediated by exaggerated TLR4 signaling on the intestinal epithelium, the onset of NEC leads to mobilization of an endogenous TLR4 ligand, high mobility group box 1 (HMGB1) from the intestine to the lungs and brain where TLR4 activation on the pulmonary epithelium and microglia respectively leads to phenomena such as neutrophil infiltration, reactive oxygen species (ROS) buildup, and other downstream effects that exacerbate pathology in the lungs, brain and other organ systems.

In full-term neonates, TLR4 expression in the intestine is notably downregulated around the time of delivery and then slowly increases as the immune system matures (66, 67). It follows that full-term neonates are protected from NEC compared to those that are born preterm, since TLR4 activation in the intestinal epithelium has been shown to be critical for NEC development (68) (**Figure 1**). TLR4 activation results in increased intestinal epithelial death by both apoptosis (69) and necroptosis (70), the induction of ER stress (71) and enhanced autophagy (72–74). Other critical features of TLR4 activation in the intestine include decreased mucosal healing both *in vitro* and *in vivo* (67), reduced goblet cell production and thus loss of a mucin barrier for defense (68), increased expression of proinflammatory cytokines leading to increased barrier permeability, decreased tight junction function, and increased epithelial cell apoptosis (75). These direct effects of TLR4 activation cumulatively promote bacterial translocation (76), which leads to further TLR4 activation on the endothelium, leading to loss of endothelial nitric oxide synthase (eNOS) and associated vasoconstriction (77), which in turn contributes to the development of ischemic necrosis in NEC. The premature intestine, which has increased expression of TLR4, is therefore more susceptible to NEC due to the factors described above (64, 65, 78).

It is important to note that other TLRs have been implicated in NEC pathogenesis. TLR5 and TLR9, particularly, have been demonstrated to be protective in NEC pathogenesis. On the other hand, TLRs 2 and 8 have been demonstrated to be upregulated in NEC intestinal tissue from experimental animal models, as well as those samples obtained in the clinical setting from infants. However, functional pathways related to the role of these latter TLRs in pathogenesis have not been clearly defined (79, 80). Similarly, other pattern recognition receptors including nucleotide‐binding domain and leucine‐rich repeat containing (NLR) proteins, RIG-I-like receptor and antimicrobial mediators such as mannose-binding lectin are upregulated in NEC and studies are ongoing to elucidate the associated pathways in order to identify viable therapeutic targets (81–84). Interestingly, emerging data suggests that one such mediator, NLR pyrin domain containing 3 (NLRP3) likely acts *via* a TLR4 dependent pathway (83).

## Mucosal Dysbiosis and Associated Disruptions in Immune Function

It has been long established that the intestinal microbiome, consisting of many millions of microbes inhabiting the gut, has a significant impact on disease processes (24). Normally, bacteria that compose the microbiome synthesize several mediators including lipopolysaccharides, peptidoglycans, short-chain fatty acids (SCFA), signaling molecules such as nitric oxide and essential vitamins which influence host physiology by regulating the mucosal immune system (85, 86). Preclinical data derived from studies in germ-free mice have shown that lymphoid follicles in the gut require peptidoglycan from gram negative bacteria for maturation, reflecting the importance of bacterial colonization for immune development (87). Other studies have shown that bacteria-naïve mice have fewer T cells and Paneth cells (88), lower expression of secretory IgA, lower levels of intestinal epithelial cells compared to conventional mice.

From clinical data, premature infants have been described to have an immature microbiome, with amniotic fluid, placenta, and meconium of premature neonates containing variations in microbes compared to full-term infants (89–91). The specific characteristics of the immature microbiome include well-described characteristic progressions from one dominant microbial population to the next (92). However, the preterm microbiome overall remains consistently less diverse compared to the variations in microbial classes observed in full term neonatal stool (92–94). For instance, in one 16S rRNA study, four bacterial classes accounted for more than 90% of the microbial sequence reads derived from preterm infant stool (92, 95). Other studies have verified lower alpha-diversity in preterm stool microbiota compared to full-term controls (96, 97). The immature preterm microbiome has also been demonstrated to impact metabolism and metabolomics, resulting in a metabolic state analogous to fasting, in spite of adequate caloric intake. Hence, metabolic derangements likely present another mediator of the relationship between microbiota disruption in preterm infants and functional immune deficiencies, which may persist and impact long-term health (98). Other factors that modulate the microbiota of premature infant include the use of antibiotics and h2-blockers, the administration of breast milk, and practices within the NICU (98–101).

Microbial dysbiosis is considered to play important roles in the complex etiology of NEC (92, 102, 103). Alterations in the microbiome have the ability to cause disruptions in immune function and result in a shift in balance to pathogenic bacterial colonization (104, 105). Further, activation of TLR4 by LPS present on potentially pathogenic gram-negative bacteria is an important aspect of NEC pathogenesis as discussed earlier. The tendency towards pathogenic microbial colonization results in TLR4 mediated phagocytosis and translocation of these Gram-negative bacteria across the intestinal mucosal barrier (106), resulting in activation of NF-κB and caspases, which triggers inflammation (107).

Overall, similar bacterial species have been identified in infants diagnosed with NEC compared to control infants. However, neonates with NEC have been described to have microbiomes that are temporally dynamic (with more changes in composition over time), but feature lower variations of strains within species (108, 109). Preterm infants with relatively lower alpha-diversity have higher risk of a later propensity for NEC development (95, 97). More specifically, 16S rRNA and metagenomic sequencing studies have reported an association between phylum *Proteobacteria* overrepresentation and increased NEC incidence (95, 110). Of note, this phylum consists of gram-negative pathogens that express high levels of LPS and are overproducers of SCFA including propionic acid which is thought to exacerbate NEC-associated neurodevelopmental conditions including movement disorders, seizure, and developmental delay (111).

While these data are compelling, reports outlining the constitution of NEC and non-NEC associated microbiota continue to vary, and further studies are needed to shed more light on this field. Ongoing studies examining how specific human milk oligosaccharides (HMOs) exert a protective effect on neonates against development have also focused in part on the microbiome. HMOs may promote the growth of bacteria from phyla Bacteroidetes, the latter of which is mainly represented by the *Bifidobacterium* genus (112, 113). These bacteria have been demonstrated to have a beneficial effect on immunity and are associated with positive outcomes with respect to NEC, as well as respiratory complications in neonates (112, 114). For this reason, probiotic therapy remains an active research area in NEC therapeutics. The influence of breast milk derived HMOs on the airway microbiome in preterm infants and its potential effects in reducing airway inflammation for instance, also remains an open question under investigation.

## *In Utero* Influences and Immune Tolerance

Various studies reveal that there are important maternal effects on immune system development during the *in utero* period (103, 115, 116). Ongoing work continues to establish that important events and influences may be exerted during this time that precede post-natal influences such as breastmilk, enteral feeds and antibiotic exposure (117–119). The maternal microbiome and related factors such as maternal diet, antibiotic use and maternal infections all remain active areas of research to find potential mechanisms to guide development of targeted interventions that can impact early colonization patterns in infants to prevent early pathology such as NEC as well as longer term diseases that may persist into adulthood (120).

One such study has demonstrated that transiently exposing germ-free mice to *E. co*li during pregnancy results in pups with increased intestinal type 3 innate lymphoid cells (ILC3) and F4/80 mononuclear cells (121), an effect that persisted 8 weeks postpartum. This effect was associated with altered intestinal transcriptional profiles and increased production of epithelial antibacterial peptides compared to those offspring from germ-free dams. Similarly, a pig model in which chorioamnionitis was induced by LPS administration resulted in higher intestinal endotoxin and neutrophil/macrophage density, with shorter villi in the offspring, accompanied by upregulation of various innate immune response, neutrophil chemotaxis and antigen processing genes after 5 days (122). Unfortunately, this and other existing studies are limited, in that they do not evaluate longer term outcomes of these alterations in the immune system. These studies also are limited by not accounting for the specific site of LPS signaling (immune versus epithelial). A longer term study has been performed in a murine model of colitis where developing mice were exposed *in utero* to LPS (123). Subsequent chemical induction in adulthood, at age 5 weeks, revealed protection from development of colitis in those pups exposed to endotoxin during development. Notably, this protection was not present when mice received LPS postnatally at 7 days of age.

While a preponderance of the studies described above involve animals, important data from human infants has also been obtained. Studies that are able to be performed without harm to infants include stool analyses in preterm neonates, which have yielded robust metabolomic data (114) and include those studies that have compared preterm neonates with NEC to healthy controls to demonstrate that NEC does not have a uniform microbial signature. Combined with emerging laboratory tools and approaches that are able to probe regulation of the immune system at the molecular level, it is becoming clearer that the preterm immune system possesses in-built immune tolerance, which is associated with impaired metabolism as described earlier, including minimized glycolytic activity (124). Accordingly, preterm naive CD4^+^ T cells have been found to have a higher threshold for inducing inflammation compared to adults, with impaired early Th1 differentiation including IFNγ production (125). Instead, Th2 and Th17 polarization are noted following bacterial stimulation, accompanied by low innate antiviral type 1 interferon responses (79, 126, 127). Similarly, IFNγ production by stimulated naive cord blood CD4^+^ T cells has been measured as 5 to 10-fold less relative to adult CD4^+^ T cells, resulting in a characterization of the preterm CD4 response as Th2 skewed (128). This allows baseline prioritization of energy for replenishment and maintenance of organ functions over inflammatory responses until a specific threshold is exceeded, as seen in the mucosal and systemic perturbations characteristic of NEC (129). This hyper-inflammatory status has been correlated with the diagnostic phase of NEC and sepsis in preterm infants (114).

## Epithelial Barrier Dysfunction

One of the direct consequences of the local inflammation caused by TLR4 activation is destruction of the intestinal epithelial barrier (130, 131). Under physiologic conditions, epithelial cells regulate the penetration of gut lumen contents including bacteria and their components, digestive enzymes and degraded food products such as ions, nutrients and water (132). This is accomplished by the formation of tight junctions containing complexes of proteins which bind the epithelial cells together (133), as well as active transport mechanisms (134, 135). A disruption of these tight junctions allows unregulated traverse of pathogenic antigens which trigger mucosal injury and inflammation leading to disorders such as NEC.

In the premature neonate, control of luminal contents is complicated by a pre-disposition to intestinal epithelial injury (25). It has been shown that proteins that make up the tight junction, including claudins and occludins, show a tissue-specific distribution pattern including those patterns characteristic of GI tract epithelium (136, 137). Further, some tight junction proteins have downregulated expression in preterm infants (138, 139), resulting in alterations in intestinal tight junction function (25, 140, 141). In one animal study, intestinal tissue obtained from germ free mice colonized by microbiota from preterm infants was found to express lower levels of occludin and tight junction associated protein ZO-1 compared to controls, in addition to disorganization in TJ protein assembly (139). Such imbalances of tight junctions are thought to render the epithelium even more susceptible to injury during NEC, during which expression of these tight junction proteins are significantly impaired *via* alterations to HIF-1 pathway signaling (142).

When TLR4 is activated during NEC, enterocyte migration from the crypt to the villus can also be impaired *via* modifications to the cell-extracellular matrix interactions (143, 144). Whereas TLR4 activation in the adult leads to increased enterocyte proliferation, in the premature intestine, apoptosis is instead induced, leading to reduced cell proliferation, impaired epithelial regeneration (69, 144, 145), and dysfunction in the epithelial barrier. In addition, autophagy – a mechanism by which cells recycle their intracellular organelles but which can also lead to cell death - is induced in intestinal epithelial cells as a consequence of TLR4 activation during NEC, leading to impaired migration (146). Strategies to block enterocyte autophagy, including epidermal growth factor therapy, have been found to reduce NEC severity in animal models (73, 147).

Interestingly, a unique pathway to epithelial injury mediated by neutrophils has been described in murine models. Neutrophil infiltration has long been noted histologically in murine and human tissue samples as characteristic of NEC pathology (148, 149). While it has been hypothesized that neutrophil recruitment may contribute to epithelial injury in NEC *via* the release of toxic products and reactive oxygen species, definitive evidence from murine models suggests that the formation of neutrophil extracellular traps (NETosis) are important actors in neutrophil-mediated epithelial damage and pathogenesis (150).

## Patient Outcomes Following Inflammatory Disorders of Premature Birth

### Gut

The immunopathology of NEC presents clinically as a devastating intestinal disease of high mortality and morbidity, with associated complications including bowel necrosis and perforation, leading to intestinal resections and resulting nutritional disorders such as short bowel syndrome. These sequelae may notably also have effects that last beyond the acute period of illness, affecting nutritional status, susceptibility to other illnesses and developmental delay. Premature infants who survive NEC are at risk of long-term severe growth failure in the years following infection (151). A number of multicenter cohort studies have showed that premature infants that underwent surgery for NEC were more likely to have significant growth delay compared to those without NEC (152, 153). This is thought to be mainly due to impaired nutritional delivery and loss of functional gastrointestinal mass secondary to bowel resection (154). However, it has been demonstrated that neonatal sepsis in premature infants also results in a hypermetabolic state with increased energy expenditure and protein catabolism that persists beyond the acute period of illness (155–157). This is important, considering that even premature infants with medical NEC are at increased risk for growth failure. Importantly, adult and animal studies have demonstrated that TNF-α, IL-1β and IL-6, which are globally elevated in sepsis and are expressed downstream of TLR4 signaling in NEC, are likely modifiers of protein and energy metabolism (158, 159). Hence while the molecular mechanisms that mediate this phenomenon are yet to be fully elucidated, it is clear that the devastating effects of exaggerated TLR4 signaling in the premature infant gut persists beyond the acute period of illness. Notwithstanding, clinical studies reviewing long-term growth impairments and gastrointestinal sequelae of NEC beyond three years are lacking. This is the case for longer term laboratory studies examining longer term immune manifestations, as previously outlined.

### Brain

Recent clinical and experimental studies have established that NEC is not only an intestinal condition (160, 161). Its broader sequelae including systemic inflammation, hypoxia, ischemia have the ability to trigger multisystem organ dysfunction, notably in the brain and lungs (162). Systematic reviews have noted that NEC is an independent risk factor for neurodevelopmental delay and poor neurocognitive outcomes in preterm infants (3, 163, 164). Importantly, these deficits may continue to manifest, with children continuing to have poor mental and psychomotor development around 2 years and in a significant proportion, persistent cognitive deficits well into school age (153, 165, 166). Proposed mechanisms for these sequalae suggest a multifactorial etiology. For instance, characteristic changes to the microbiome associated with NEC affect the not only gut, but may also mediate preterm brain development *via* the modulation of neurotransmitter levels (167). As in the gut, endothelial barrier dysfunction in the brain is a critical aspect of NEC pathophysiology, as the blood-brain-barrier (BBB) has been found to be directly impacted by dysbiosis. In this case, mouse models have demonstrated that low levels of SCFA due to alterations in bacterial composition can cause a permanent increase in BBB permeability due to alterations in tight junction protein expression (168).

Most broadly, downstream intestinal injury and barrier dysfunction discussed in prior sections promote bacterial translocation into systemic circulation, accompanied by the recruitment of inflammatory mediators and cytokines, leading to systemic inflammation and sepsis. The brain’s own immune cells, the microglia and astrocytes, modulate the development of normal brain functions including synaptic pruning, synapse formation and synaptic transmission regulate neurogenesis, neuronal migration, and synaptic plasticity (169). However, multiple animal studies have demonstrated that exposure to inflammatory molecules disrupts brain development. For instance, exposure to IL-1β and TNF-α in one mouse study resulted in long‐lasting disruptions in oligodendrocyte maturation (170). In other animal studies, early exposure to LPS has resulted in reduction in hippocampal volume, disordered neurogenesis, increased microglia populations and activity, axonal injury, and memory impairment (171, 172). Accordingly, increased disease severity, for instance, surgical NEC (173) and increased levels of pro-inflammatory cytokines (160), are associated with worse neurodevelopmental outcomes in preterm infants.

Finally, it is now known that TLR4 signaling is an important part of NEC pathophysiology in the brain (17). Using a clinically relevant murine NEC model, Nino et al. showed that TLR4 activation occurs during NEC, an endogenous TLR4 ligand, high mobility group box 1 (HMGB1) is released from the intestinal and activates TLR4 on microglia, leading to the accumulation of reactive oxygen species (ROS), loss of oligodendrocyte progenitor cells (OPCs), dysmyelination, and cognitive impairments. Importantly, the administration of targeted microglia anti-inflammatory and antioxidant therapy ameliorated the degree of neurological dysfunction, demonstrating a novel therapeutic target in NEC-associated brain injury.

### Lung

Extra-intestinal sequelae of NEC are well documented in the pulmonary system. Up to half of premature infants born prior to 36 weeks gestation develop lung injury (174). However, lung injury that occurs in the presence of infectious and inflammatory neonatal conditions such as NEC tend to be more severe and have longer term effects than in matched patients without (12). Overall, approximately 15% of infants with NEC experience lung damage that is characterized by neutrophil infiltration and inflammatory cytokine upregulation.

Interestingly, recent insights into underlying causes of NEC-induced lung injury have revealed a mechanism mediated by TLR4 that is analogous to that seen in brain injury (175, 176). Using a murine NEC model, Jia et al. have shown that TLR4, highly expressed on the pulmonary epithelium in animals with NEC, is activated by TLR4 ligand high-mobility group box 1 (HMGB1) derived from the gut epithelium. This leads to downstream upregulation of CXCL5, a chemoattractant, and subsequent recruitment of neutrophils. Importantly, the aerosolized delivery of a novel TLR4 small molecule inhibitor was sufficient to reverse this inflammatory cascade and prevent the lung injury normally triggered by NEC in this model. Further, in NEC and other diseases with similar histopathology, Th17 skewed CD4 T cells have been shown to drive the cytokine upregulation and immune cell infiltration that constitute the mechanism of inflammation and lung injury (79, 177). The inflammation is typically *via* upregulation of the chemokine CCL25, which is now known to be upregulated downstream of TLR4 activation in the lung during NEC, a phenomenon that simultaneously depletes the population of protective regulatory T cells (Tregs) present in the lung epithelium (176).

## Summary

The premature neonate has multiple levels predispositions to infectious and inflammatory afflictions such as NEC. A dynamic and complicated interface between pathogens, other microbiota, the maternal environment and the immune system mediates such pathology and has severe acute effects locally at the intestinal epithelium, but importantly impacts multiple systems secondary to systemic inflammation, as well as widespread effects of important mediators such as TLR4. Short term disruptions to intestinal cell renewal and homeostasis, metabolism and growth, gut microbial colonization, neurodevelopment, and lung physiology are evident from clinical studies. Longer term data are largely deficient from even clinical literature. Yet, a growing field of NEC and associated research continue to shed light on how the above discussed monumental disruptions in the immune system impact long term immune function and how knowledge gleaned from such phenomena can play a role in prevention and treatment of such neonatal disorders.

## Author Contributions

All authors listed have made a substantial, direct, and intellectual contribution to the work and approved it for publication.

## Funding

DJH is supported by grants from the National Institutes of Health, USA under award numbers R01 DK1117186 and R01 DK121824.

## Conflict of Interest

DH is supported by research grants from Abbott Nutrition and Noveome.

The remaining authors declare that the research was conducted in the absence of any commercial or financial relationships that could be construed as a potential conflict of interest.
